# Recovery of ferrous sulfate from basic oxygen furnace sludge using waste sulfuric acid

**DOI:** 10.1016/j.mex.2026.103960

**Published:** 2026-05-15

**Authors:** Bilguun Mend, Jang-Ho-Jay Kim, Yong-Sik Chu

**Affiliations:** a Climate and Energy R&D Group, Korea Institute of Ceramic Engineering and Technology, Jinju 52581, Republic of Korea; b School of Civil and Environmental Engineering, Yonsei University, Seoul 03722, Republic of Korea

**Keywords:** Basic oxygen furnace sludge, Waste sulfuric acid, Ferrous sulfate, Hydrometallurgical recycling, Cyclic filtration, Industrial waste valorization, Resource recovery

## Abstract

Basic oxygen furnace (BOF) sludge and waste sulfuric acid are industrial waste streams that require effective management due to environmental and disposal concerns. In this study, a laboratory-scale hydrometallurgical method was developed to recover ferrous sulfate from BOF sludge using waste sulfuric acid (50% purity). The method integrates controlled acid leaching, low-temperature crystallization, and cyclic reuse of the recovered filtrate. The key features of the protocol are:

• Selective acid leaching of Fe-bearing phases from BOF sludge using waste sulfuric acid at 70 °C
• Low-temperature crystallization of hydrated ferrous sulfate phases at 5 °C
• Cyclic reuse of the recovered filtrate to improve solution efficiency

The protocol was validated through repeated leaching–crystallization cycles. X-ray diffraction analysis confirmed the dissolution of Fe-bearing phases from the BOF sludge and the formation of hydrated ferrous sulfate phases, including melanterite, rozenite, and szomolnokite, in the recovered products. Average ferrous sulfate yields of 8.86 ± 0.22 g, 10.10 ± 0.18 g, and 10.60 ± 0.26 g were obtained for Cycles A, B, and C, respectively, corresponding to estimated Fe recovery efficiencies of 40.2%, 46.8%, and 51.4%. These results demonstrate that the proposed method provides a reproducible route for converting BOF sludge and waste sulfuric acid into value-added ferrous sulfate while enabling filtrate reuse.

## Graphical abstract

**Figure:**
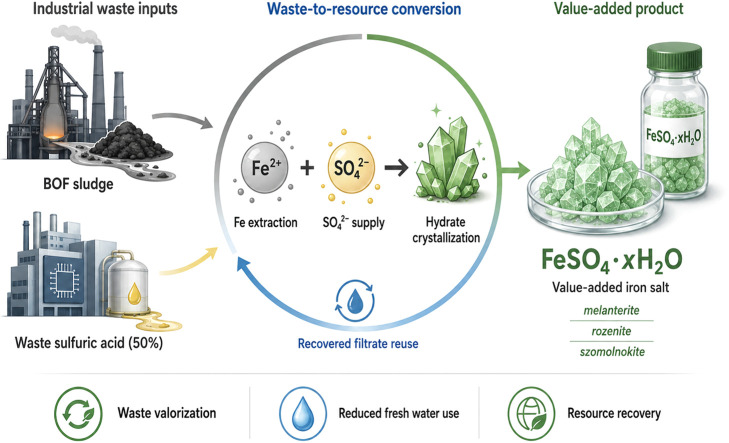

Specifications table

**Table:** 

**Subject area**	Materials Science
**More specific subject area**	Industrial waste recycling
**Name of your method**	Recycling of waste-derived ferrous sulfate
**Name and reference of original method**	Acid leaching and crystallization of metal sulfate salts
**Resource availability**	BOF sludge, waste sulfuric acid, filtration unit, drying oven

## Background

Basic oxygen furnace (BOF) sludge is a by-product generated during steelmaking and typically contains a high proportion of iron together with minor metal impurities [1]. Large quantities of BOF sludge are produced worldwide, and its management presents environmental and regulatory challenges. Conventional disposal options such as landfilling or stabilization lead to resource loss and may create long-term environmental risks [2].

At the same time, waste sulfuric acid is generated in significant quantities from various industrial processes, particularly from semiconductor wafer cleaning operations [3]. In semiconductor fabrication, sulfuric acid–based solutions such as sulfuric peroxide mixtures (SPM, H₂SO₄–H₂O₂) are widely used to remove photoresist and organic residues from silicon wafers [4,5]. After repeated use, these cleaning solutions become spent and are discharged as sulfuric-acid-rich waste streams that require treatment before disposal.

Hydrometallurgical processes based on acid leaching have been investigated to recover iron from steelmaking residues. However, many reported methods rely on fresh acid consumption and single-pass leaching systems, which generate secondary liquid waste and reduce overall resource efficiency. The present work describes a laboratory-scale method for recovering ferrous sulfate from BOF sludge using waste sulfuric acid derived from semiconductor cleaning processes. The protocol integrates controlled acid leaching, low-temperature crystallization, and cyclic reuse of the leaching filtrate to enable iron recovery while reducing liquid waste generation.

## Method details

### Overview of the recycling protocol

**Fig. 1:**
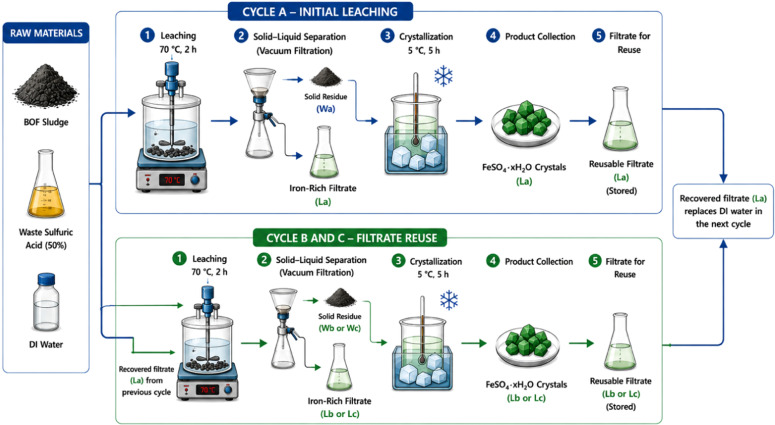
Schematic representation of the laboratory-scale process for recovering FeSO₄·xH₂O from basic oxygen furnace (BOF) sludge using waste sulfuric acid. Cycle A represents the initial leaching–crystallization process using freshly diluted waste sulfuric acid, while Cycles B and C represent subsequent reuse cycles in which the recovered filtrate replaces deionized water in the leaching solution. FeSO₄·xH₂O crystals were recovered independently from each cycle, and the remaining filtrate was collected for reuse in the following cycle.

Fig. 1 illustrates the schematic workflow of the laboratory-scale process developed for recovering hydrated ferrous sulfate (FeSO₄·xH₂O) from basic oxygen furnace (BOF) sludge using waste sulfuric acid (50% purity). The protocol consists of three sequential stages:

1. acid leaching of BOF sludge
2. solid–liquid separation followed by low-temperature crystallization
3. cyclic reuse of the recovered filtrate. In this protocol

Cycle A represents the initial leaching–crystallization process using freshly diluted waste sulfuric acid, whereas Cycles B and C represent subsequent reuse cycles in which the recovered filtrate from the previous cycle replaces deionized water in the next leaching solution. FeSO₄·xH₂O crystals are recovered independently from each cycle, and the remaining filtrate is collected for reuse in the following cycle. This configuration enables the recovery of iron as ferrous sulfate while reducing freshwater consumption and minimizing secondary liquid waste generation.

### Raw materials

Basic oxygen furnace (BOF) sludge obtained from an integrated steel plant was used as the iron-bearing precursor for ferrous sulfate synthesis. Prior to use, the sludge was air-dried to reach a constant weight and then gently ground to obtain a homogeneous powder. The elemental composition of the BOF sludge was determined using inductively coupled plasma optical emission spectroscopy (ICP-OES), and the results are summarized in Table 1. The analysis confirmed that iron is the dominant component in the sludge, together with smaller amounts of Ca, Zn, Mn, and other elements typically present in steelmaking residues. Waste sulfuric acid (50% purity) originating from semiconductor wafer cleaning processes was used as the acidic leaching medium. Deionized water was used for dilution and washing where required.

**Table 1:** Elemental composition of basic oxygen furnace (BOF) sludge determined by inductively coupled plasma optical emission spectroscopy (ICP-OES). Values are expressed in wt.% (mean ± standard deviation, n = 3).

Compound	SiO_2_	Al₂O₃	Fe₂O₃	CaO	MgO	P₂O₅	Na₂O	K₂O	MnO	ZnO	LOI
BOF sludge	1.10	0.50	82.20	6.17	1.12	0.52					
	0.26	0.21	0.58	6.00	0.01						

### Experimental setup

Leaching experiments were conducted in a 200 mL glass reaction vessel placed on a temperature-controlled heating plate and mixed using a magnetic stirrer operating at 200 rpm. Solid–liquid separation was performed using a vacuum filtration unit with paper filters. Crystallization was carried out in a laboratory refrigerator maintained at approximately 5 °C, and the recovered crystals were dried in a drying oven at temperatures not exceeding 40 °C.

## Recycling process

The recycling protocol for recovering ferrous sulfate from BOF sludge using waste sulfuric acid consisted of sequential leaching, solid–liquid separation, and low-temperature crystallization steps, followed by reuse of the filtrate in subsequent cycles.

### Step 1. Acid leaching

In a typical batch, 40 mL of waste sulfuric acid (50% purity) was mixed with 60 mL of deionized water to prepare the leaching solution. Then, 10 g of BOF sludge was gradually added to the diluted acidic solution. The suspension was heated to 70 °C and maintained for 2 h to promote dissolution of iron-bearing phases present in the BOF sludge, resulting in an iron-rich leachate.

### Step 2. Solid–liquid separation

After completion of the leaching step, the suspension was subjected to vacuum filtration to separate the insoluble solid residue from the iron-containing solution. The residue was washed with a small amount of deionized water to recover entrained solution. The combined filtrate was collected for the subsequent crystallization stage.

### Step 3. Crystallization and product recovery

The filtrate obtained from the leaching step was cooled to approximately 5 °C, within a practical low-temperature range of 3–8 °C and maintained for about 5 h to induce crystallization of hydrated ferrous sulfate (FeSO₄·xH₂O). This temperature range was selected based on preliminary observations: excessively low temperatures may cause partial freezing of the solution, whereas higher temperatures slow down supersaturation development and delay crystallization. Thus, 5 °C was used as the target crystallization temperature because it effectively promotes ferrous sulfate hydrate formation while avoiding freezing and remaining achievable using a conventional laboratory refrigerator.

### Selection of operating conditions

The operating conditions were selected based on preliminary laboratory observations. For acid leaching, tests were conducted at 60–80 °C with reaction times of 1–5 h. Among the tested conditions, 70 °C provided the highest ferrous sulfate yield with the lowest amount of residual solid, while 2 h was selected as an energy-efficient reaction time that allowed sufficient Fe dissolution without unnecessarily extending the process duration. For crystallization, the filtrate was cooled to approximately 5 °C, within a practical low-temperature range of 3–8 °C. This range was selected because excessively low temperatures may cause partial freezing of the solution, whereas higher temperatures slow supersaturation development and delay crystallization. Therefore, the selected leaching and crystallization conditions were chosen to balance Fe dissolution, product recovery, energy efficiency, and practical laboratory operation.

### Filtrate reuse

After crystallization, the recovered filtrate was collected and reused in subsequent cycles. The pH of the recovered filtrate after crystallization was approximately 5, indicating that its acidity had decreased during the leaching–crystallization process. Therefore, for each reuse cycle, 60 mL of recovered filtrate was mixed with 40 mL of fresh waste sulfuric acid to restore the acidity of the leaching solution to approximately pH 0–1. In this process, the recovered filtrate replaced deionized water as the dilution medium, while the newly added waste sulfuric acid supplied sufficient acidity for further Fe dissolution. Under the present laboratory conditions, the recovered filtrate was reused for two subsequent cycles after the initial batch. The comparable product yields obtained in Cycles A–C indicate that this filtrate reuse strategy did not significantly reduce ferrous sulfate recovery within the tested reuse range. However, because the pH, sulfate concentration, dissolved Fe content, and impurity levels of the recovered filtrate may gradually change during extended reuse, routine monitoring and adjustment of filtrate chemistry are required to determine the maximum number of reuse cycles for long-term operation.

## Method validation

The proposed recycling protocol was validated through phase analysis of both the solid residues and the recovered crystalline products, as well as by evaluating the consistency of the product yield across repeated cycles.

**Fig. 2:**
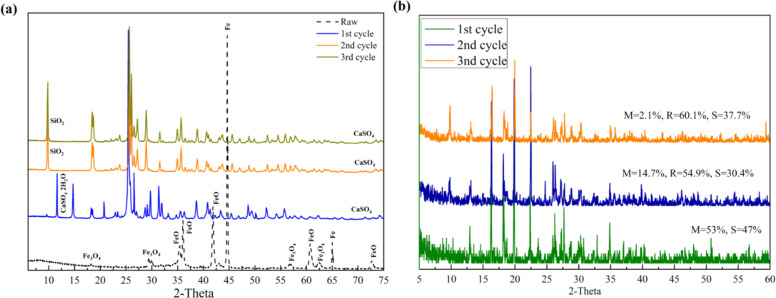
X-ray diffraction (XRD) patterns obtained during validation of the recycling protocol. (a) XRD patterns of the raw BOF sludge and the solid residues obtained after the 1st–3rd leaching cycles, showing the dissolution of iron-bearing phases and the enrichment of CaSO₄ in the remaining solids. (b) XRD pattern and Rietveld refinement of the recovered ferrous sulfate product, indicating the presence of hydrated ferrous sulfate phases including melanterite (FeSO₄·7H₂O), rozenite (FeSO₄·4H₂O), and szomolnokite (FeSO₄·H₂O).

Fig. 2a shows the X-ray diffraction (XRD) patterns of the raw BOF sludge and the solid residues obtained after the first, second, and third leaching cycles. The diffraction patterns indicate that the iron-bearing phases present in the raw BOF sludge were progressively dissolved during the leaching process. As the leaching cycles proceeded, the intensity of iron-related peaks decreased, while the remaining residues became enriched in calcium sulfate (CaSO₄). This observation confirms that iron contained in the BOF sludge was effectively transferred from the solid phase into the solution during the acid leaching step. The crystalline product obtained after the crystallization stage was further characterized by XRD analysis. As shown in Fig. 2b, the diffraction pattern together with the Rietveld refinement results indicates that the recovered product mainly consists of hydrated ferrous sulfate phases. The identified phases include melanterite (FeSO₄·7H₂O), rozenite (FeSO₄·4H₂O), and szomolnokite (FeSO₄·H₂O), confirming that the dissolved iron was successfully recovered as FeSO₄·xH₂O during the crystallization stage.

Quantitative phase analysis based on XRD/Rietveld refinement was used to evaluate the phase transformation during leaching and crystallization. In the solid residues, the relative contribution of Fe-bearing phases decreased after acid leaching, while CaSO₄ became increasingly dominant, indicating preferential dissolution of Fe-containing components from the BOF sludge. In contrast, the recovered crystalline product was mainly composed of hydrated ferrous sulfate phases, including melanterite (FeSO₄·7H₂O), rozenite (FeSO₄·4H₂O), and szomolnokite (FeSO₄·H₂O). These results confirm that Fe-bearing phases in the BOF sludge were effectively dissolved during the leaching step and subsequently recovered as FeSO₄·xH₂O during low-temperature crystallization.

The reuse strategy was evaluated by comparing the initial cycle with the subsequent recovered-filtrate cycles. In Cycle A, the leaching solution was prepared using deionized water as the dilution medium, whereas in Cycles B and C, deionized water was replaced by recovered filtrate. Although the recovered filtrate had a higher pH after crystallization, its acidity was restored before each reuse cycle by adding fresh waste sulfuric acid. The consistent yields summarized in Table 2 indicate that this pH-adjusted recovered-filtrate reuse strategy maintained sufficient acidity and dissolved species for FeSO₄·xH₂O crystallization within the tested cycles. Together with the XRD/Rietveld results, which confirmed the decrease of Fe-bearing phases in the residues and the formation of hydrated ferrous sulfate phases in the recovered products, these results demonstrate that the proposed recycling protocol provides reproducible Fe dissolution and recovery during repeated cycles.

**Table 2:** Ferrous sulfate yield during repeated recycling cycles. Values are shown as mean ± standard deviation based on 10 runs.

Cycle	Leaching medium	Adjusted pH	Yield of FeSO₄·xH₂O (g)	CV (%)	n
Cycle A	WSA + DI water	0–1	8.86 ± 0.22	2.5	10
Cycle B	WSA + recovered filtrate	0–1	10.10 ± 0.18	1.8	10
Cycle C	WSA + recovered filtrate	0–1	10.60 ± 0.26	2.5	10

Note: Before each reuse cycle, 60 mL of recovered filtrate was mixed with 40 mL of fresh waste sulfuric acid to adjust the leaching solution to approximately pH 0–1. CV = coefficient of variation.

To further evaluate the recovery performance, a product-based Fe recovery efficiency was estimated from the recovered FeSO₄·xH₂O yield and the initial Fe content of the BOF sludge. Because direct Fe concentrations in the leachate and solid residues were not measured, the calculated values represent estimated recovery efficiencies rather than absolute Fe extraction rates. The initial Fe mass in the BOF sludge was calculated from its Fe₂O₃ content using Eq. (1):

(1) $$\text{mFe},\text{initial}\;=\;\text{msludge}\;\times \;\text{wF}e_{2}O_{3}\;\times \;\left(2\text{MFe}\;/\;\text{MF}e_{2}O_{3}\right)$$

where msludge is the mass of BOF sludge used, wFe₂O₃ is the Fe₂O₃ mass fraction in the BOF sludge, and MFe and MFe₂O₃ are the molar masses of Fe and Fe₂O₃, respectively. Based on 10 g of BOF sludge containing 82.20 wt.% Fe₂O₃, the initial Fe mass was approximately 5.75 g. The Fe recovery efficiency was then estimated using Eq. (2):

(2) Ferecoveryefficiency(%)=(mFe,recovered/mFe,initial)×100

where mFe, recovered was estimated from the recovered FeSO₄·xH₂O product yield and the hydrated ferrous sulfate phase composition identified by XRD/Rietveld refinement. Based on this product-based mass balance, the estimated Fe recovery efficiencies were approximately 40.2%, 46.8%, and 51.4% for Cycles A, B, and C, respectively. These values indicate that recovered-filtrate reuse-maintained Fe recovery performance within the tested cycles. Nevertheless, future work should include direct leachate and residue chemistry to establish a complete Fe mass balance and determine absolute Fe extraction efficiency.

## Conclusions

This study developed a laboratory-scale recycling protocol for recovering hydrated ferrous sulfate from BOF sludge using waste sulfuric acid. The proposed method integrates acid leaching at 70 °C, solid–liquid separation, low-temperature crystallization at 5 °C, and recovered-filtrate reuse. XRD/Rietveld analysis confirmed the decrease of Fe-bearing phases in the leached residues and the formation of hydrated ferrous sulfate phases, including melanterite, rozenite, and szomolnokite, in the recovered products. Repeated cycles produced stable ferrous sulfate yields, demonstrating the reproducibility of the proposed protocol. The recovered-filtrate reuse strategy, combined with pH adjustment using fresh waste sulfuric acid, maintained recovery performance while reducing freshwater consumption.

The product-based Fe recovery efficiencies were estimated to be approximately 40.2%, 46.8%, and 51.4% for Cycles A, B, and C, respectively. These results indicate that BOF sludge and waste sulfuric acid can be converted into value-added ferrous sulfate through a simple and reproducible laboratory-scale process. Future work should focus on optimizing acid dosage, reaction time, sludge particle size, pH control, and long-term filtrate reuse. Direct leachate and residue chemistry should also be included to establish a complete Fe mass balance and to evaluate the scalability of the process for industrial application.

## Limitations

The proposed recycling protocol was developed and validated at a laboratory scale, and its performance may vary under larger-scale operational conditions. The chemical composition of BOF sludge and waste sulfuric acid can differ depending on the industrial source, steelmaking process, and operating conditions, which may influence acid consumption, Fe dissolution efficiency, impurity accumulation, and FeSO₄·xH₂O crystallization behavior. Therefore, in practical applications, key process parameters such as acid dosage, liquid-to-solid ratio, reaction time, leaching temperature, and recovered-filtrate reuse conditions should be optimized according to the Fe content, acid concentration, pH, sulfate concentration, and impurity levels of the input materials.

The pH of the recovered filtrate may also change during repeated reuse. Therefore, pH adjustment before leaching is required to maintain sufficient acidity for Fe dissolution, and routine monitoring of filtrate chemistry is necessary to ensure stable recovery performance. In addition, prolonged exposure of the leachate to air may promote partial oxidation of Fe²⁺ to Fe³⁺, potentially affecting crystallization behavior and the phase distribution of hydrated ferrous sulfate products. Future work should include direct leachate and residue chemistry, full Fe mass-balance analysis, long-term recovered-filtrate reuse tests, and pilot-scale validation to evaluate the scalability and robustness of the proposed method.

## Related articles

Mend B. et al. (2025). Utilisation of industrial sludge-derived ferrous sulfate for hexavalent chromium mitigation in cement. Advances in Cement Research. https://doi.org/10.1680/jadcr.24.00239.

## For a published article

Mend B. et al. (2026). Performance and environmental assessment of Portland cement incorporating waste-derived ferrous sulfate as a gypsum substitute: A case study. Case Studies in Construction Materials. https://doi.org/10.1016/j.cscm.2026.e05909.

## CRediT authorship contribution statement

**Bilguun Mend:** Conceptualization, Methodology, Writing – original draft, Writing – review & editing, Visualization, Validation. **Jang-Ho-Jay Kim:** Supervision, Validation. **Yong-Sik Chu:** Supervision, Validation, Funding acquisition.

## Declaration of competing interest

The authors declare that they have no known competing financial interests or personal relationships that could have appeared to influence the work reported in this paper.

## Data Availability

Data will be made available on request.
